# Looking for the inflexion point of the Frank-Starling curve

**DOI:** 10.1186/cc12144

**Published:** 2013-03-19

**Authors:** HD Aya, M Geisen, C Ebm, N Fletcher, M Grounds, A Rhodes, M Cecconi

**Affiliations:** 1 St George's Healthcare NHS Trust, London, UK

## Introduction

Fluid responsiveness is defined based on an arbitrary increase of cardiac output (CO) or stroke volume (SV) of 10 to 15%. We hypothesise that the variation of heart efficiency (Eh) and the slope (S) defined by the relative increase of CO over the relative increase of mean filling pressure (Pmsa) can be used as alternative definitions of fluid responsiveness.

## Methods

Patients admitted to the ICU were monitored with a calibrated LiDCOplus (LiDCO, UK) and Navigator (Applied Physiology, Australia) to estimate Pmsa and Eh (Pmsa -central venous pressure/Pmsa). A 250 ml fluid challenge was performed over 5 minutes. Categorical data were compared by Pearson chi-square test. Correlation was assessed by Kappa test. The inflexion point of S to define responders was obtained by ROC curve analysis.

## Results

**Figure 1:**
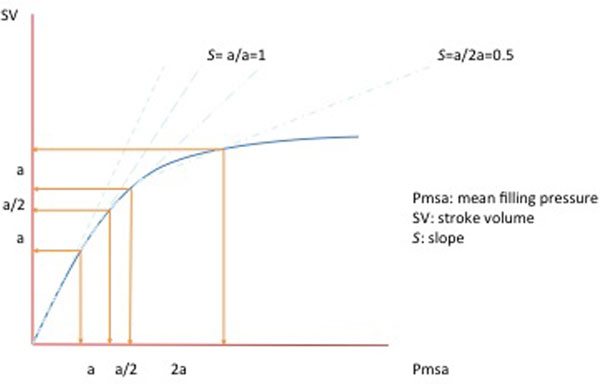


A total of 104 fluid challenges were observed in 40 patients. ROC curve analysis reveals an area under the curve of 0.93 (95% CI = 0.85 to 1, *P <*0.001). The best cutoff or the slope was 0.76 (sensitivity 0.92, specificity 0.93). The proportions of responders identified by the ΔEh (Table 1) and by the slope method (Table 2) are smaller compared with the relative increase of SV method. Significant correlation was found between both methods and the ΔSV (ΔEh κ = 0.54, *P <*0.001; S κ = 0.55, *P <*0.001). See Figure 1.

**Table 1:** Distribution of events according to ΔSV and ΔEh

	Response by ΔSV ≥10%	Response by ΔEh ≥0	*P *value
Nonresponder	62 (59.6%)	76 (73.1%)	<0.001
Responder	42 (40.4%)	28 (26.9%)	<0.001

**Table 2:** Distribution of events according to ΔSV and the slope (S)

	Response by ΔSV ≥10%	Response by S ≥0	*P *value
Nonresponder	62 (59.6%)	75 (72.1%)	<0.001
Responder	42 (40.4%)	29 (27.9%)	<0.001

## Conclusion

Moderate agreement is observed between new and current definitions of fluid responsiveness.
